# Basicervical femoral neck fractures: an observational study derived from the Swedish Fracture Register

**DOI:** 10.2340/17453674.2024.40503

**Published:** 2024-05-22

**Authors:** Jonas SUNDKVIST, Per HULENVIK, Viktor SCHMIDT, Per JOLBÄCK, Mikael SUNDFELDT, Per FISCHER, Cecilia ROGMARK, Hans JUTO, Olof WOLF, Sebastian MUKKA

**Affiliations:** 1 Department of Diagnostics and Intervention (Orthopaedics), Umeå University, Umeå; 2 Institute of Clinical Sciences, Sahlgrenska Academy, Gothenburg University, Gothenburg; 3 Department of Orthopaedics, Institute of Clinical Science, The Sahlgrenska Academy, Gothenburg University, Gothenburg; Department of Research, Development, Education and Innovation, Skaraborg Hospital, Skövde; 4 Faculty of Medicine and Health, Örebro University, Örebro; Department of Orthopedics, Karlstad Central Hospital, Karlstad, Region Värmland; 5 Department of Orthopaedics, Lund University, Skåne University Hospital, Malmö; 6 Department of Surgical Sciences, Orthopaedics, Uppsala University, Uppsala, Sweden

## Abstract

**Background and purpose:**

Limited research has been conducted on basicervical femoral neck fractures (bFNFs). The importance of displacement in clinical outcomes remains unclear. We aimed to characterize patient demographics, degree of displacement, treatment, treatment failures, and reoperations in a cohort of fractures from the Swedish Fracture Register (SFR).

**Methods:**

1,260 fractures in 1,185 individuals ≥ 60 years who had a bFNF registered in the SFR at 6 orthopedic departments from 2011 to 2020 were screened through radiographic review. The final sample included 291 patients with a confirmed bFNF. The medical records of these 291 patients were reviewed. We assessed baseline characteristics, initial fracture dislocation, treatment methods, tip–apex distance, failures, reoperations, and mortality.

**Results:**

The mean age was 82 years (range 60–101, 55% women). 98 (34%) were undisplaced and 193 (66%) displaced. All patients underwent operative treatment. In the undisplaced group 95 (97%) patients received internal fixation (IF) and 3 (3%) had primary hip arthroplasty. In the displaced group 149 (77%) received IF and 41 (21%) had primary hip arthroplasty. 33 (11%) suffered treatment failure. When treating an undisplaced bFNF with IF, only 3 (3%) experienced treatment failure, in contrast to the 24 (16%) failure rate for a displaced bFNF.

**Conclusion:**

Undisplaced bFNFs have a low failure rate when treated with IF. For displaced bFNF treated with IF the failure rate is considerably higher. There is a need for further investigation of classification, treatment, and outcome of bFNF.

The basicervical femoral neck fracture (bFNF) (Figure 1) affects the base of the femoral neck where it connects to the trochanter and is defined as a 2-part fracture immediately medial to the intertrochanteric line [1]. John R. Moore first described this fracture type in 1939 [2]. In contrast to other hip fractures, there is little research on bFNFs. The lack of studies might be partially due to the elusive nature of the bFNF. First, at different times, it has been described as an extracapsular and intracapsular fracture [1,3]. Second, the bFNF does not have a unique ICD-10 code. Third, the degree of displacement is not accounted for in the Arbeitsgemeinschaft fur Osteosynthesfragen Foundation/Orthopaedic Trauma Association (AO/OTA) classification [1]. Displacement is a known risk factor for complications and failures in transcervical femoral neck fractures treated with internal fixation (IF) [4-6]. The proportion of bFNFs has been reported to range from 1.8–12% of all femoral neck fractures (FNFs] [7-10]. According to the Norwegian Hip Fracture Register, bFNFs account for 4.6% of all FNFs [11]. IF treatment with cannulated screws, sliding hip devices (SHDs), intramedullary nails (IMNs), and hip arthroplasty has been proposed [9,12-15]. Studies on bFNFs are uncommon and limited to case series and smaller cohorts. Hence, there is a need for a multicenter cohort study. Using data from the Swedish Fracture Register (SFR), we aimed to outline the demographics, displacement levels, treatment methods, treatment failures, and reoperations in a cohort of bFNFs.

**Figure 1:**
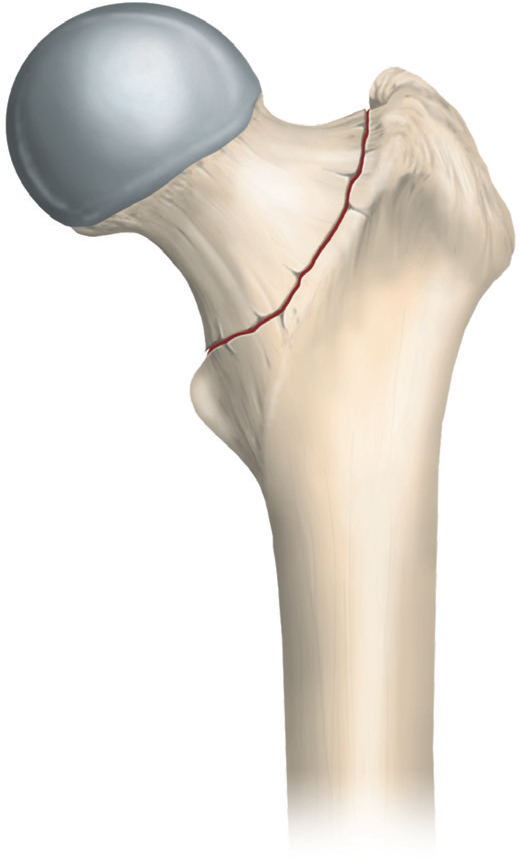
Graphic representation of a bFNF.

## Methods

### Study design and setting

Data for this observational cohort study was sourced from the SFR at 6 participating departments (Gothenburg/Mölndal, Karlstad, Sunderbyn, Umeå, Uppsala, and Östersund) [16]. Our report complies with the Strengthening the Reporting of Observational studies in Epidemiology (STROBE) guidelines. Since its introduction in 2011, the SFR has served as a national quality register for managing fractures and their treatment. Data on individuals who sustain a fracture in Sweden is collected based on the unique permanent Swedish 12-digit personal identification number (PIN) [17]. The data includes injury location, mechanism of injury, and fracture classification, mostly through the 2007 AO/OTA system [18]. Through the prespecified web-based interface, data on treatment and subsequent reoperations is also registered by the treating surgeon. The registration of femoral fractures in the SFR has shown high accuracy and validity [19]. Compared with the Swedish National Patient Register, the SFR had a femoral fracture completeness rate of 81% in 2022 [20].

**Figure 2:**
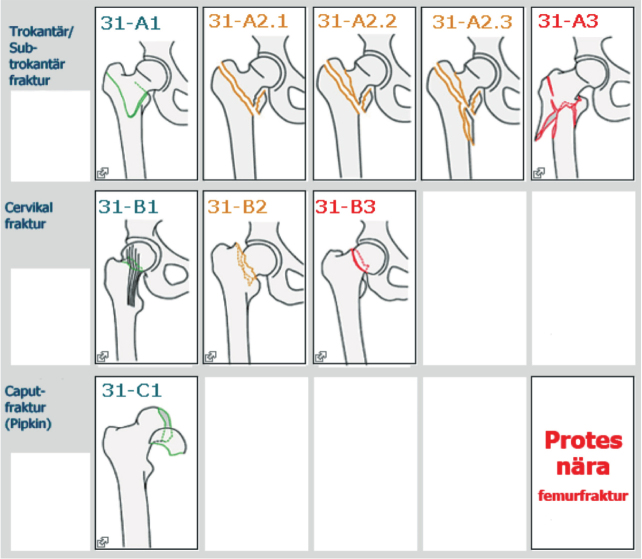
Classification of hip fractures as it appears in the SFR (in Swedish).

Through stepwise implementation, the SFR’s coverage has significantly improved from 40% in 2014 to full coverage (100%) in all 54 orthopedic departments in Sweden by 2021. The registration of FNFs in the SFR uses a modified version of the AO/OTA classification 2007 (Figure 2, see Appendix) [18]. It includes undisplaced or minimally displaced FNFs (SFR 31-B1), displaced FNFs (SFR 31-B3), and bFNFs (SFR 31-B2). In addition to information on high- and low-energy trauma, the register collects data on stress, spontaneous, and pathological fractures. Treatment is registered as either nonoperative or operative. Operative treatment is further specified to include type of fixation (screws or pins, SHD, long and short IMNs, anatomic plates), hip arthroplasty (hemi [HA] or total [THA], cemented or cementless fixation), or other (i.e., excision arthroplasty).

### Patients

Patients aged ≥ 60 years, registered in the SFR between 2011 and 2020 with a bFNF (SFR 31-B2), were screened at the 6 participating orthopedic departments. Participating sites were chosen for access to medical records and radiographs (authors’ departments) and their completeness (2022) of hip fractures (ICD S.72) in the SFR (Gothenburg/Mölndal 89%, Karlstad 94%, Sunderbyn 89%, Umeå 90%, Uppsala 90%, and Östersund 88%) [21].

Plain anteroposterior and lateral radiographs were used to verify taxonomy according to the AO/OTA classification modified by the SFR; these images were also reviewed if computed tomography (CT) or magnetic resonance imaging (MRI) was available. Only fractures with the main fracture zone immediately medial to the intertrochanteric line were included. Transcervical FNFs were excluded (n = 649). If the fracture extended laterally past the intertrochanteric line, it was classified as trochanteric (n = 219). Fractures not immediately apparent were discussed by the authors (JS, VS, PF, PH, MS OW, HJ, SM) and only included if all agreed. We excluded fractures with low-quality radiographs where the primary fracture zone was not clearly visible. The use of CT and MRI for diagnosis was documented. The degree of displacement was classified as undisplaced and displaced fractures. Tip–apex distance (TAD) was measured on plain postoperative radiographs [21].

### Data collection

We used the PIN to collect data, including the review of medical records of all contributing departments, to verify and ensure the completeness of the data. Patient data included age, sex, American Society of Anesthesiologists (ASA) classification, cognitive impairment (none, suspected, definitive), pre-fracture walking ability (no difficulty, with aid, not at all), admission from sheltered housing (yes/no), initial treatment, any hip-related complication, and reoperations. Patients were followed up for at least 2 years or until death, whichever occurred first.

### Outcome measurements

Outcome measures included treatment failure, defined as nonunion, peri-implant fracture, avascular necrosis (AVN), posttraumatic osteoarthritis (defined as the occurrence of new radiographic signs of osteoarthritis in combination with clinical symptoms involving hip joint pain), and surgical site infection (SSI). Other outcome measures included reoperations, defined as implant removal or adjustment of osteosynthesis, secondary hip arthroplasty, excision arthroplasty, or re-osteosynthesis due to subsequent fractures around the implants and surgical debridement, antibiotics, and implant retention (DAIR) due to SSI. Closed reduction of a dislocated hip arthroplasty was documented. Treatment failures not resulting in reoperation were also reported.

Mortality rates were documented at 30 days, 90 days, and 1 year.

### Statistics

Variables are presented as absolute numbers and proportions of all fractures. Nominal variables are presented as proportions of all fractures and scale variables as median, range, or mean and standard deviation (SD). Relative risk including 95% confidence intervals (CI) was calculated to compare treatment failures between undisplaced and displaced bFNF. CI was calculated for the treatments given and treatment failures. All statistical analyses were performed using the SPSS software (IBM SPSS Statistics for Mac, Version 26.0, IBM Corp, Armonk, NY, USA).

### Ethics, data sharing, funding, use of artificial intelligence, and disclosures

The study complied with the ethical principles of the Helsinki Declaration and was approved by the Swedish Ethical Review Authority (2022-06685-01).

The dataset used in this study is not publicly accessible to protect patient data privacy. We are positive to sharing data but are legally restricted from sharing the data publicly according to the law on Public Access and Secrecy, chapter 21, paragraph 7 and chapter 25, paragraph 1 (https://www.riksdagen.se/sv/dokument-lagar/dokument/svensk-forfattningssamling/offentlighets--och-sekretesslag-2009400_sfs-2009-400). Those interested in the data set can contact the corresponding author at Umeå University to discuss data sharing in compliance with Swedish laws, or can also apply directly to the Center of Registers, Västra Götaland (URL: http://registercentrum.se/), but approval is needed from the Swedish Ethical Review Authority.

The study was funded by grants from the regional Agreement on Medical Training and Clinical Research (ALF) between Västerbotten County Council and Umeå University and between Skåne Region and Lund University. This work was supported by the Department of Orthopaedics, Umeå University Hospital. No artificial intelligence tools were used to analyze data or write the present study. The authors declare no potential conflicts of interest for the research, authorship, or publication of this article. Complete disclosure of interest forms according to ICMJE are available on the article page, doi: 10.2340/17453674.2024.40503

## Results

### Patients and descriptive data

**Figure 3:**
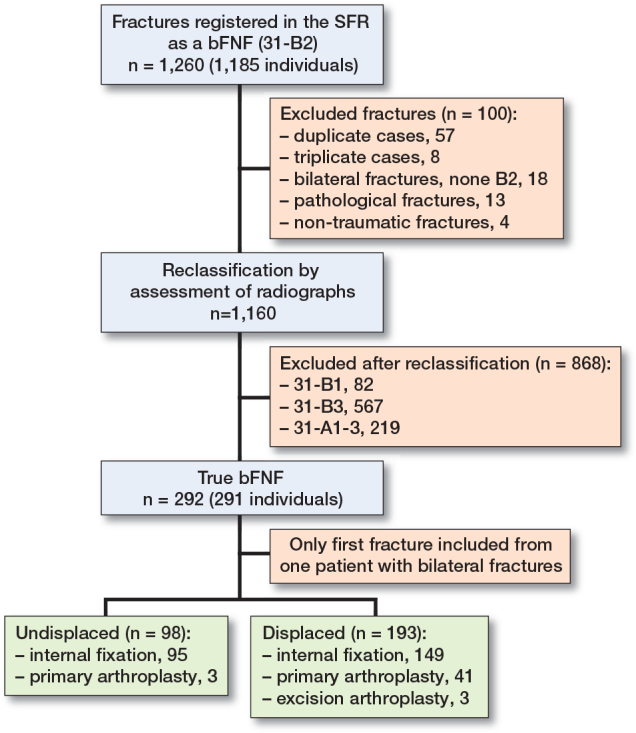
Patient flowchart.

1,260 bFNFs in 1,185 patients were extracted from the SFR. Of these, 868 fractures were excluded after reviewing radiographs (Figure 3). The final sample comprised 291 patients with 291 bFNFs. Each department contributed: Gothenburg/Mölndal 134, Karlstad 36, Sunderbyn 29, Umeå 36, Uppsala 36, and Östersund 20 patients. The mean age was 82 (SD 9, range 60–101) years and 55% were women (Table 1). The median follow-up was 35 (range 0–137) months. Cognitive dysfunction was classified as suspected or definitive in 102 (35%) patients and 84 (29%) were admitted from sheltered housing. 69 patients were ASA 1–2 (24%) and 131 were ASA 3–5 (45%). ASA classification was missing in 91 (31%) patients. Falls from standing (73%) and falls from lower than standing (9%) (i.e., bed or chair) in or near the residence were the most common causes of injury (Table 1).

**Table 1:** Patient characteristics (N = 291). Values are frequency and (%) unless otherwise specified

Mean age (range)	82 (60–101)
Women	159 (55)
ASA classification ^a^	
1–2	9 (24)
3–5	131 (45)
Missing	91 (31)
Cognitive impairment	
None	185 (64)
Suspected	38 (13)
Definitive	64 (22)
Missing	4 (1.4)
Walking ability	
Unassisted	112 (39)
With an aid	154 (53)
Not at all	21 (7.2)
Missing	4 (1.4)
Sheltered housing	84 (29)
Injury cause	
Simple fall	211 (73)
Fall due to ice/snow	15 (5.2)
Fall from height	1 (0.3)
Fall from furniture	26 (8.9)
Fall from wheelchair	7 (2.4)
Fall from stairs	6 (2.1)
Bicycle	3 (1.9)
Unspecified fall	22 (7.6)

a ASA = American Society of Anesthesiologists.

### Fracture classification

Of all bFNFs, 98 (34%) were undisplaced and 193 (66%) displaced. CT and/or MRI were used as adjuncts to diagnose 28 patients (10%).

### Treatment

All patients underwent primary surgical treatment (Table 2). In the undisplaced group 95 (97%, CI 91–99) patients were treated with IF and 3 (3%, CI 0.6–8.7) with HA. In the displaced group 149 (77%, CI 71–83) patients received IF and 41 (21%, CI 16–28) hip arthroplasty, divided into 25 cemented HA, 13 cemented THA, and 3 hybrid THA (cemented stem, uncemented cup). 3 (2%, CI 0.3–4.5) patients underwent primary excision arthroplasty.

**Table 2:** Treatment options for all bFNF fractures and undisplaced and displaced fracture subgroups. Values are frequency and (%)

	Undisplaced bFNFs (n = 98)	Displaced bFNFs (n = 193)	All bFNFs (n = 291)
Sliding hip screw	28 (29)	41 (21)	69 (24)
Sliding hip screw + anti-rotation screw	17 (17)	25 (13)	42 (14)
Twinhook (sliding hip device)	46 (47)	62 (32)	108 (37)
Intramedullary nail	4 (4.1)	21 (11)	25 (9)
Hip hemiarthroplasty (cemented)	3 (3.1)	25 (13)	28 (10)
Total hip arthroplasty (cemented)	0 (0)	13 (6.7)	13 (4.5)
Total hip arthroplasty (hybrid)	0 (0)	3 (1.6)	3 (1.0)
Excision arthroplasty	0 (0)	3 (1.6)	3 (1.0)

The TAD of all patients treated with IF was measured, revealing a mean and median of 19 mm (SD 6, range 6–40).

### Treatment failures and reoperations

During a minimum follow-up of 2 years, treatment failure occurred in 33 patients (11%, CI 7.9–16). When treated with IF, 3 patients (3%, CI 0.7–9.0) experienced treatment failure following an undisplaced bFNF; the corresponding number for those with a displaced bFNF was 24 (16%, CI 11–23) (relative risk 5.1, CI 1.6–16) (Table 3). 17 of the failures in the IF group led to conversion to hip arthroplasty. 5 patients underwent reoperation due to SSI, all following treatment of a displaced bFNF. Additionally, 4 patients in the IF group had documented hip joint pain without discernible signs of treatment failure on plain radiographs, resulting in 1 conversion to THA and 3 implant removals.

**Table 3:** Treatment failures (n = 54) of internal fixation stratified by fracture subtype and re-operations. Values are frequency and (%)

	Undisplaced bFNFs (n = 95)	Displaced bFNFs (n = 149)	All bFNFs (n = 244)	Reoperation (n = 23)
All treatment failures	3 (3.1)	24 (16)	27 (11)	
Avascular necrosis	0 (0)	2 (1.3)	2 (0.8)	1 implant removal,
				1 THA
Cut-out	0 (0)	7 (4.7)	7 (2.9)	7 THAs
Non-union	2 (2.1)	8 (5.4)	10 (4.1)	7 THAs
Peri-implant fracture	0 (0)	1 (0.7)	1 (0.4)	1 HA
Surgical site infection	0 (0)	5 (3.4)	5 (2.0)	2 excision arthroplasty,
				3 DAIRs
Posttraumatic arthritis	1 (1.1)	1 (0.7)	2 (0.8)	1 THA

Of patients treated with primary arthroplasty, 6 (14%, CI 5.1–27) had treatment failure: 2 dislocations (treated with closed reduction), 2 SSIs (treated with DAIR), and 2 periprosthetic femur fractures (treated with IF).

### Mortality

Of the 291 patients, 26 (9%) died within 30 days, 53 (18%) within 90 days, and 84 (29%) within 1 year.

## Discussion

We aimed to characterize patient demographics, degree of displacement, treatment, treatment failures, and reoperations in a cohort of fractures from the SFR.

We found that treating undisplaced bFNFs with IF has a low failure rate (3%). For displaced bFNF (two-thirds of all), when treated with IF the failure rate was 5 times higher.

**Figure 4:**
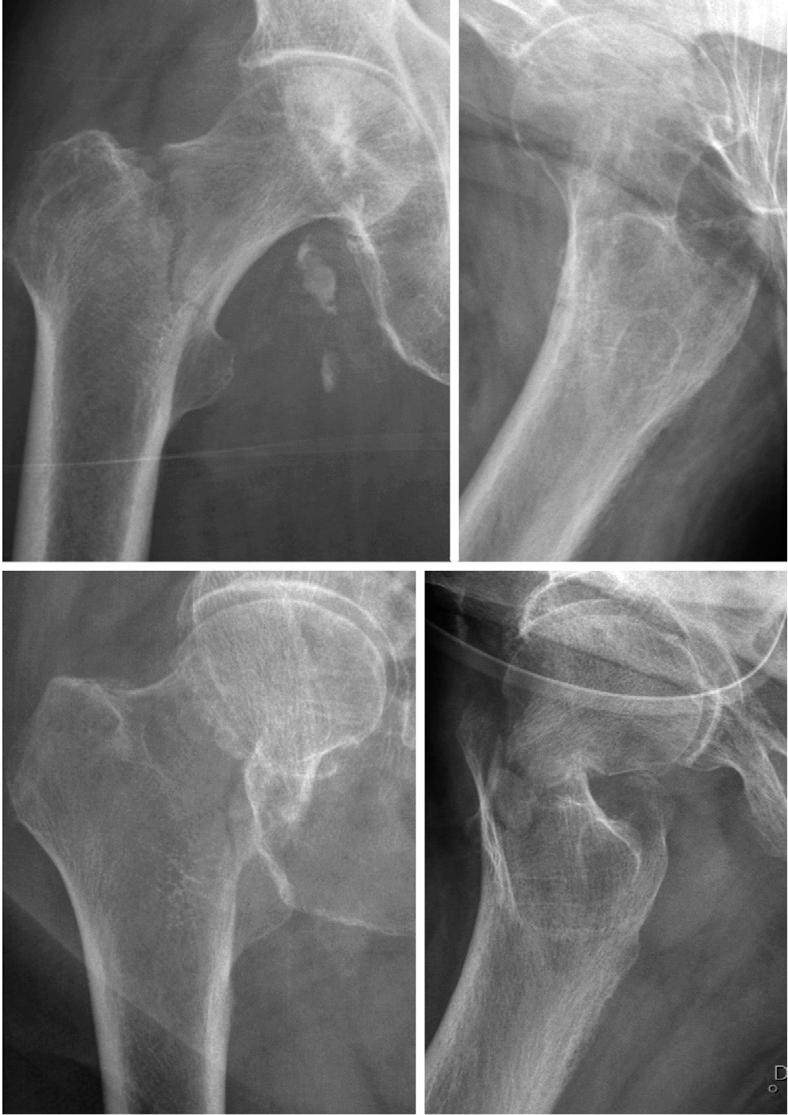
Antero-posterior and lateral radiograph of a true bFNF (top) and a displaced transcervical FNF (bottom). The lateral radiograph reveals the femoral neck attached to the proximal fragment (top) and distal fragment (bottom).

Our findings are similar when comparing our demographic data with other studies on FNFs [22-24]. The low rate of authentic bFNFs suggests that classifying them accurately is an arduous task. The base of the femoral neck is often hidden by the trochanter on an externally rotated hip joint. Thus, a thorough analysis of the lateral radiograph is often essential to accurately identify and diagnose an authentic bFNF (Figure 4, see Appendix) [7]. More liberal use of CT for correct diagnosis, as suggested by Dekhne et al., seems reasonable [13]. We are not aware of any studies that validate the bFNF classification. Our group previously reported a higher proportion of bFNFs, but this was based on unverified register data [10]. Through careful analysis of the radiographs, the participants found a lower rate of true bFNFs.

The treatment options reported in this study suggest a lack of agreement and that the current evidence is insufficient to guide clinicians [13]. Nevertheless, various IF and primary hip arthroplasty methods have shown satisfactory outcomes [9,15]. With a fracture line at the base of the femoral neck, the relationship between the joint capsule with its associated blood supply and the proximal fragment is unclear. Optimizing treatment of bFNFs could involve addressing technical (i.e., fracture reduction, implant choices, and implant positioning) and biological factors (i.e., whether the bFNF is an intra- or extracapsular fracture or both), as well as individual/institutional algorithms or treatment traditions [25,26].

The reoperation rate after IF for a bFNF in our cohort is consistent with the findings of Mallick and Parker and Chen et al., which are the largest cohorts presented so far [8,24]. These findings contradict the higher complication rate documented by Watson et al. [27]. Such results suggest that bFNFs are positioned between transcervical FNFs (intracapsular) and trochanteric fractures (extracapsular), both anatomically and complication-wise [5,21,28,29]. Moreover, we observed a relatively high complication rate in patients undergoing primary arthroplasty compared with Davis et al., whose data suggests that hip arthroplasty is a valid treatment option in displaced bFNFs [15].

### Limitations

Our study design incorporated the SFR to obtain a sizable cohort consisting of more than 1,200 bFNFs. Our decision was based on the high accuracy and validity of other femoral fractures registered in the SFR [19]. However, the assessment of radiographs resulted in the exclusion of a large proportion of fractures. The final sample size prevented us from analyzing factors associated with treatment failures and reoperations. The classification of bFNFs is difficult in a national register such as the SFR, calling for updated user instructions. Furthermore, the observational study design reflects different local treatment regimes, including variations in follow-up. Also, the lack of functional outcomes could mask complications. Finally, some factors affecting the treatments offered to patients could not be extracted from the medical records.

The major strength of our study lies in its large cohort of authentic bFNFs, incorporating pre- and postoperative radiographic analysis, clinical outcomes presented as treatment failures, and subsequent reoperations.

### Conclusion

We found that treating undisplaced bFNFs with IF has a low failure rate (3%). For displaced bFNF treated with IF the failure rate was 5 times higher. Both IF and arthroplasty are offered in clinical practice and have proven effective. Our findings suggest that undisplaced bFNFs can be treated effectively with a fixed-angle implant during IF. Optimal treatment for displaced bFNFs is still unknown, and large multicenter studies that include patient-reported outcomes could provide further guidance in managing bFNFs.

## Notes

JS initiated the study, collected data, performed radiographic measurements, conducted statistical analyses, and wrote the manuscript. PH, VS, MS, PF, and HJ collected data, performed radiographic measurements, and wrote the manuscript. OW and CR initiated the study, wrote and reviewed the manuscript. PJ collected data and wrote and reviewed the manuscript. SM initiated the study, supervised JS, performed statistical analysis, and wrote and reviewed the manuscript.

The authors would like to thank the Swedish Fracture Register administrative staff. They are grateful to all the physicians and secretaries at the orthopedic departments in Sweden for their invaluable contributions in managing the SFR. The authors thank Pontus Andersson (Pontus Art Production AB) for illustrating a bFNF (Figure 2). A special thank you is offered to all the participating patients and the valued colleagues around the country who assisted in gathering data for the present study.

Handling co-editors: Ivan Hvid and Robin Christensen

*Acta* thanks Frede Frihagen and Martyn Parker for help with peer review of this manuscript.
